# Six Chinese patients with propionic acidemia: from asymptomatic to death in the neonatal period

**DOI:** 10.1186/s13023-025-03622-6

**Published:** 2025-03-12

**Authors:** Shunan Wang, Lulu Li, Yulan Ma, Haihe Yang, Yuting Sang, Yue Tang, Lifei Gong, Jinqi Zhao, Lijin Gu, Yuanyuan Kong, Xinmei Mao

**Affiliations:** 1 Department of Newborn Screening Center, Beijing Obstetrics and Gynecology Hospital, Capital Medical University, Beijing Maternal and Child Healthcare Hospital, Beijing, China; 2 Peking University First Hospital Ningxia Women and Children’s Hospital (Ningxia Hui Autonomous Region Maternal and Child Health Hospital), Yinchuan, China

**Keywords:** Propionic acidemia, *PCCA* gene, *PCCB* gene, Propionyl-CoA carboxylase, Newborn screening

## Abstract

**Background:**

Propionic acidemia (PA) is a severe organic acidemia that can result in multi-organ damage and is potentially fatal. The rarity of this disease and the limited number of reported cases contribute to a lack of comprehensive knowledge, particularly concerning the genotype-phenotype correlation. This study aims to report on PA cases in Beijing and Ningxia, China, identify the pathogenic genetic factors involved, and explore the relationship between genotype and phenotype.

**Methods:**

We calculated the positive screening rates of PA in Beijing and Ningxia and summarized data from six Chinese patients with PA identified at the Beijing Newborn Screening Center and Ningxia Newborn Screening Center. Clinical examinations included blood tandem mass spectrometry, urine gas chromatography-mass spectrometry, and the next-generation sequencing (NGS) technology. Candidate mutations were validated using polymerase chain reaction and Sanger sequencing technology. Bioinformatics software was used to analyze the pathogenicity of the variants, and Swiss PDB Viewer software was employed to predict the effect of mutations on PCCA and PCCB proteins.

**Results:**

The updated incidence of PA was 1 in 114,820 in Beijing and 1 in 189,671 in Ningxia. We reported five patients diagnosed with PA through newborn screening (NBS) and one additional patient diagnosed clinically. Among the five patients diagnosed by NBS, the two late-onset patients exhibited normal neurodevelopment, while all three early-onset patients succumbed between 4 days and 18 months of age. The patient diagnosed clinically passed away at 20 days of age. NGS showed one patient carries compound mutations in the *PCCA* gene and three patients carry compound heterozygous or homozygous mutations in the *PCCB* gene. A total of two mutations in *PCCA* (c.985T > A and c.1195 C > T) and five mutations in *PCCB* (c.1076 C > T, c.1087T > C, c.224 A > C, c.1339 C> T, and c.1033G > C)were identified, including one novel *PCCA* mutation (c.985T > A) and four novel *PCCB* mutations (c.1076 C > T, c.224 A > C, c.1339 C> T, and c.1033G > C). Bioinformatics analysis indicated these mutations are pathogenic, and Swiss PDB Viewer predictions suggest that these variations affect protein conformation.

**Conclusions:**

The updated incidence rates of PA in Beijing and Ningxia provide new epidemiological insights. We reported six patients with PA, and identified one novel mutation (c.985T > A) in *PCCA* and four novel mutations (c.1076 C > T, c.224 A > C, c.1339 C> T, and c.1033G > C) in *PCCB*, which expands the spectrum of clinical features and genetic mutations associated with PA. The c.985T > A mutation in *PCCA* and the c.1076 C > T mutation in *PCCB* may be associated with late-onset PA, while the c.224 A > C, c.1339 C > T, and c.1033G > C mutations in *PCCB* are related to early-onset PA.

## Introduction

Propionic acidemia (PA) is an organic acid disorder caused by mutations in the *PCCA* or *PCCB* genes [1]. These mutations impair propionyl coenzyme A carboxylase (PCC), leading to metabolic disorders involving propionate, propiogenic amino acids, and odd-chain fatty acids [2]. As a result, abnormal metabolites such as methylcitrate, 3-hydroxypropionic acid, and propionylglycine accumulate, causing damage to multiple organs, including the heart, brain, liver, kidneys, bone marrow and pancreas, and can be fatal [3–9]. The symptoms of PA are heterogeneous, ranging from asymptomatic to severe, depending on the degree of enzyme deficiency [10]. PA is classified as early-onset (within the first 3 months of life) and late-onset (after 3 months) [10, 11]. Previous studies have shown that early-onset PA is associated with higher mortality, whereas late-onset PA has a better prognosis, such as fewer frequencies of metabolic disorders and less neurocognitive impairment [12]. The primary treatment for PA is dietary management, which restricts propiogenic amino acids, propionate, and odd-chain fatty acids. If dietary management is insufficient to prevent severe clinical manifestations, liver transplantation is the next option [1]. The prevalence of PA varies globally. In most regions, the incidence of PA is below 1 per 100,000 newborns, except in the Middle East and North Africa [13]. In northern China, the prevalence of PA ranges from 1 in 283,459 to 1 in 3,156 [14].

In this study, we investigated six Chinese patients diagnosed at the Beijing Newborn Disease Screening Center and the Peking University First Hospital Ningxia Women and Children’s Hospital (Ningxia Hui Autonomous Region Maternal and Child Health Hospital). Five patients were diagnosed through newborn screening (NBS), and one was clinically confirmed. We analyzed their diagnostic process, treatment, and prognosis, as well as their biochemical and genetic features. Additionally, we reported the updated prevalence of PA in Beijing and Ningxia. The aim of this study was to broaden the spectrum of clinical features and gene mutations in patients with PA and to explore the relationship between phenotype and genotype in these patients.

## Materials and methods

### Newborn screening

In accordance with the “Technical Specification for Newborn Screening (2010 Edition),” heel blood was collected from newborns who had been breastfed for 72 h after birth. The samples were dried on S&S903 blood filter paper and then sent to the Beijing Newborn Screening Centre or the Ningxia Newborn Screening Centre for testing. Blood samples from 344,461 neonates in Beijing (collected between April 2014 and March 2024) and 379,341 neonates in Ningxia (collected between May 2016 and March 2024) were measured for amino acids and acylcarnitines levels using tandem mass spectrometry (MS/MS). Elevated propionylcarnitine (C3) and its ratio to acetylcarnitine (C2), free carnitine (C0), and methionine (Met) will prompt a recall for further review.

### Patients clinical features and ethics statement

The study protocol was reviewed and approved by the Medical Ethics Committee of Beijing Maternity Hospital of Capital Medical University (2022-KY-087-01). All of the guardians voluntarily participated in the study and signed informed consent forms. We studied six patients: five were diagnosed through newborn screening (three from Beijing and two from Ningxia) and one through clinical onset (from Ningxia). Among them, five were male and one was female.

### Metabolic examinations

Neonatal screening for amino acid and carnitine spectrum was conducted using tandem mass spectrometry (TQ Acquity Mass Spectrometer, Waters Corporation, USA). Clinical testing for amino acid and carnitine spectrum was performed using the NeoBase Non-derivatized MSMS Kit (PerkinElmer, Turku, Finland). For differential diagnosis of other metabolic diseases, urine organic acids were tested by gas chromatography-mass spectrometry (Shimadzu GCMS-QP2010).

### Genetic testing and bioinformatics analysis

High-throughput sequencing was performed for genetic testing. Briefly, 2 ml of blood was extracted from the proband and the parents. DNA was sequenced using the Illumina HiSeq 2500 system (Illumina, San Diego, USA). After filtering the raw data, clean data were aligned to the human genome reference sequence hg19. Mutations were compared with databases such as HGMD, gnomAD, and ExAC.

### Predictive analysis of pathogenicity

Functional prediction of missense mutations was performed by PolyPhen-2, MutationTaster, Sorting Intolerant from Tolerant (SIFT), and REVEL. Variant frequencies were determined using the 1000 Genomes Project, ExAC, and gnomAD databases. The variants were interpreted following the American College of Medical Genetics and Genomics (ACMG) 2015 guidelines.

Swiss-PDB Viewer software was used to predict and evaluate the crystal structure of the mutant proteins. The protein structures of PCCA and PCCB (PDB ID: 7YBU) were acquired from the PDB database and analyzed using Swiss-PDB Viewer 4.1.0 to visualize the protein structures and predict the effects of mutation sites on the tertiary protein structures.

## Results

### The prevalence of PA in Beijing and Ningxia

In Beijing, 344,461 neonates were tested between April 2014 and March 2024. Three patients were diagnosed with PA (Table 1), resulting in an overall incidence of 1/114,820. In Ningxia, 379,341 neonates were tested between May 2016 and March 2024. Two patients were diagnosed with PA (Table 1), with an overall incidence of 1/189,671.

**Table 1:** Basic information of the patients with PA

Case	Gender	Birth weight	Diagnosis by NBS	Age of onset	Targeted treatment	Clinical outcome	Clinical phenotype
1	Female	3000	Yes	1d	No	Died at 4d	Early-onset
2	Male	2370	Yes	-	No	Normal neurodevelopment	Late-onset
3	Male	3190	Yes	-	Yes	Normal neurodevelopment	Late-onset
4	Male	3150	Yes	3 m	Discontinued at 3 m	Died at 3 m	Early-onset
5	Male	3500	No	20d	No	Died at 40d	Early-onset
6	Male	4300	Yes	16d	Yes	Died at 18 m	Early-onset

### Clinical features of six patients with PA

#### Case 1

A female, full-term newborn (40 weeks, BW 3000 g) presented with coma and intermittent convulsions at 1 day old. Besides neonatal convulsions, she had complications including myocardial damage, renal insufficiency, hypocalcemia, neonatal hyperglycemia, neonatal jaundice, and hyperammonemia. She died at 4 days old. She was eventually diagnosed with PA. Blood for newborn screening was collected at 3 days old, and she was registered at the Beijing Newborn Screening Center for “elevated C3, C3-related ratios, and several amino acids” at 15 days old. The final diagnosis was confirmed as PA.

#### Case 2

A male, preterm newborn (36 weeks, BW 2370 g) was registered at the Beijing Newborn Screening Center at 18 days old for “elevated C3/C2 and C3/C0 on neonatal disease screening.” Repeat findings confirmed significant increases in C3 and C3/C2. He was diagnosed with PA by genetic testing, but the parents refused treatment and follow-up. The last follow-up was conducted by telephone when he was 17 months old, His weight was 12 kg (50-75th percentile) and his length was 82 cm (50th percentile). He was able to say “mama”, “dada”, and some common object names, climb stairs, and follow simple commands.

#### Case 3

A male, full-term newborn (39 weeks, BW 3190 g) was registered at the Beijing Newborn Screening Center at 20 days old for “elevated C3/C2 and decreased citrulline on neonatal disease screening.” Repeat results indicated mildly elevated C3 and C3/C2. He was diagnosed with PA and started dietary treatment at 41 days old. Starting at 2 months old, his creatine kinase (CK) and creatine kinase-MB (CK-MB) were continuously twice the upper limit of normal, indicating potential myocardial injury. He started treatment with Coenzyme Q10 (10 mg/d), fructose dipllosphate sodium (1 g/d), and levocarnitine (50 mg/kg/d). When he was 3 months old, he conducted his electrocardiogram and cardiac ultrasound, and the results were normal. The last follow-up, conducted at 10 months of age, showed his weight was 12.5 kg (> 97th percentile) and his length was 80 centimeters (> 97th percentile). His intellectual assessment results were normal, as measured by the 0–6 Years Children’s Neuropsychological Development Scale. His CK, CK-MB, electrocardiogram and cardiac ultrasound were all within normal limits. His treatment regimen included dietary management (with the ratio of special treatment milk powder to ordinary milk powder ranging from 1:2 to 1:3), coenzyme Q10 (25 mg/d), and levocarnitine (210 mg/kg/d).

#### Case 4

A male, full-term newborn (38 weeks + 4 days, BW 3150 g) was registered at the Ningxia Newborn Screening Center at 27 days old for “elevated C3 and C3/C2 on neonatal disease screening.” He was diagnosed with PA and started on dietary treatment (special treatment milk powder: ordinary milk powder = 1:2) and L-carnitine (100 mg/kg/d) at 1-month-old. After one week of treatment, his blood C3/C2 levels did not significantly change, but the associated metabolites in his urine (3-hydroxypropionic acid, propionylglycine, tiglylglycine, methylcitric acid) were significantly reduced (Tables 2 and 3). At 2 months old, his weight was 5.2 kg (25th percentile) and his length was 58.5 cm (50th percentile). He presented visual tracking and was able to lift his head. However, his treatment was discontinued, and he died at 3 months old.

**Table 2:** Blood tandem-mass spectrometry results of the patients

Case	Age	Blood tandem mass spectrometry (µmol/L)						
		C3 (0.4−5)	C3/C2 (0.03–0.2)	C3/C0 (0.02–0.27)	C3/MET (0.02–0.2)	C0 (8.6–60)	MET (10–55)	Gly (180–1000)
1	3d	10.83	0.82	1.57	0.23	6.91	47.83	650.93
2	3d	4.11	0.33	0.26	0.29	15.84	14.01	258.41
2	33d	17.86	0.57	0.53	0.78	34.02		
3	3d	3.29	0.26	0.23	0.12	14.35	28.13	373.87
3	20d	5.3	0.53	0.16	0.18	33.17	29.6	204.42
4	3d	18.65	2.33	2.66	1.43	7	13	518
4	34d	83.07	2.31	2.60	27.69	32	3	782
5	32d	17.49	6.56	1.23		14.25		
6	3d	21.56	0.71	0.97	0.92	22.29	23.31	838.94
6	11d	12.25	1.24	1.13	0.55	10.86	22.21	1225.43

**Table 3:** Gas chromatography-mass spectrometry results of the patients

Case	Age	Gas chromatography-mass spectrometry (mmol/mmol Cr)					
		Methylcitric acid (0−0.7)	Methylcitric acid−4 (0−0.8)	3-Hydroxypropionic acid−2 (0.0–4.0)	Propionylglycine (0−0.4)	Tiglylglycine−1(0.0–5.0)	Tiglylglycine−2(0.0−0.5)
3	20d	2.05	2.6	7.32	0	0.15	0
3	2 m	1.49	1.86	5.56	0	0.18	0
4	27d	39.2	39.2	107.8	16.5	17.6	156.2
4	34d	34.2	34.2	29.8	2.9	2.4	0
5	32d	8.7	8.7	79.7	15.5	7.5	NA
6	11d	29.7	20.7	193.8	8.7	34.7	8.6

#### Case 5

A male, full-term newborn (BW 3500 g) from a consanguineous marriage presented with drowsiness and feeding difficulties from 24 days old after a fever. He was taken to a local hospital. He had complications of pyemia, protein-energy malnutrition, hypoimmunoglobulinemia, neonatal anemia, thrombocytopenia, atrial septal defect, electrolyte disturbance (hyponatremia, hypocalcemia), and metabolic acidosis with respiratory alkalosis. He died at 40 days old. He was diagnosed with PA by blood and urine metabolic tests.

#### Case 6

The younger brother of Case 5, a male, full-term newborn (38 weeks + 4 days, BW 4300 g), was registered at the Ningxia Newborn Screening Center at 11 days old for “significantly elevated C3 and related ratios on neonatal disease screening.” Since his brother was suspected to have died of an inherited metabolic disease, he started dietary treatment (special: regular milk powder = 2:1), L-carnitine (250 mg/kg/d), and hydroxocobalamin (1 g/d) at 11 days old before diagnosis. At 16 days old, he showed poor feeding and a lack of mental alertness. His blood ammonia was 196 µmol/l. EEG suggested mildly abnormal activity, and cranial ultrasound indicated slightly enhanced white matter echoes. Cranial MRI suggested abnormal signals in several brain regions. His treatment was adjusted to include arginine (0.9 g/d) and sodium benzoate (0.9 g/d). His clinical symptoms improved, and ammonia levels normalized. At 18 months of age, following a respiratory tract infection, he developed metabolic acidosis again, accompanied by type 1 respiratory failure. Despite active treatment, including acid correction, infection control, and respiratory support, the last arterial blood gas analysis showed a pH of 6.94, PCO2 of 83.3 mmHg, and lactate levels that were too high to measure. He passed away after emergency interventions were discontinued.

### Metabolic examinations

All patients except Case 5 were diagnosed with NBS. All five patients showed elevated C3/C2 during NBS. C3 and C3/C0 were variably elevated. All cases showed elevated C3, C3/C2, and C3/C0 upon re-check (Table 2). Except for Case 3, who had elevated 3-hydroxypropionic acid but normal propionylglycine, all other patients exhibited elevated propionylglycine, 3-hydroxypropionic acid, tiglylglycine, and methylcitric acid (Table 3).

### Gene sequencing results

**Fig. 1:**
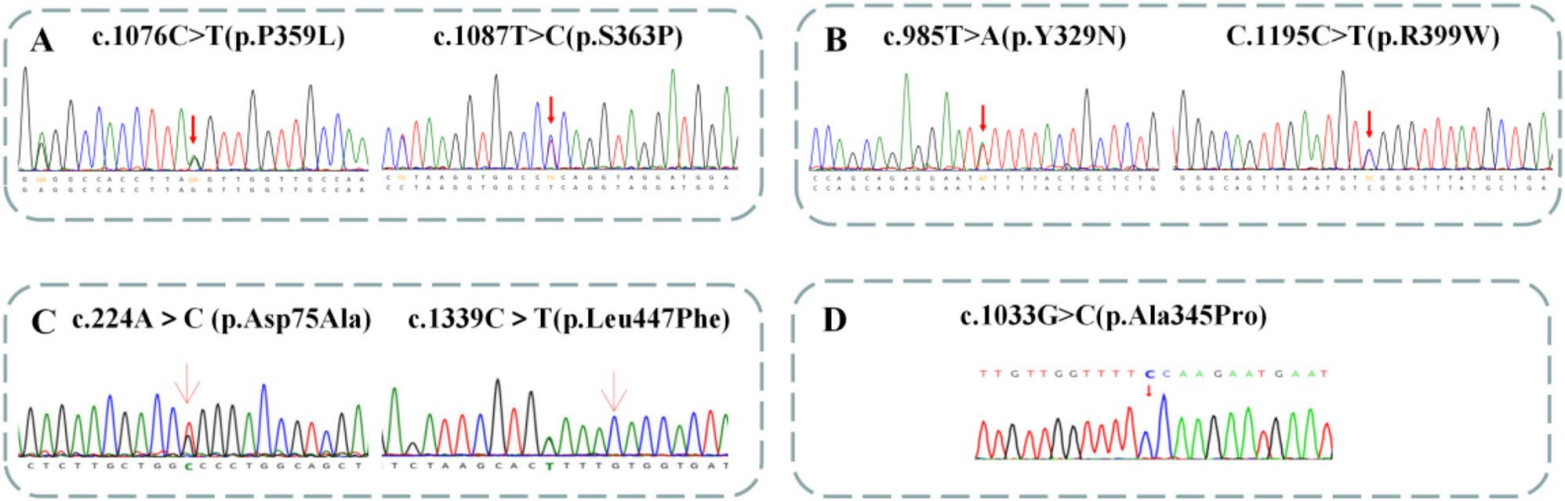
Available genetic testing analysis of patients with PA. (**A**) In case 2, sanger sequencing revealed the patient had the compound heterozygous variants c.1076 C > T (p.P359L) of paternal origin and c.1087T > C (p.S363P) of maternal origin in the *PCCB* gene. (**B**) In case 3, sanger sequencing revealed the patient had the compound heterozygous variants c.985T > A (p.Y329N) of paternal origin and c.1195 C > T (p.R399W) of maternal origin in the *PCCA* gene. (**C**) In case 4, sanger sequencing revealed the patient had the compound heterozygous variants c.224 A> C (p.Asp75Ala) of paternal origin and c.1339 C> T (p.Leu447Phe) of maternal origin in the *PCCB* gene. (**D**) In case 6, the patient had the compound homozygous variant c.1033G > C (p.Ala345Pro) in the *PCCB* gene

Four patients did genetic tests. Next-generation sequencing revealed that one patient carries mutations in the *PCCA* gene, while three patients carry mutations in the *PCCB* gene. Specifically, the genetic test identified two mutations in the *PCCA* gene (c.985T > A and c.1195 C > T) and five mutations in the *PCCB* gene (c.1076 C > T, c.1087T > C, c.224 A > C, c.1339 C > T, and c.1033G > C). Among these, one novel mutation (c.985T > A) was found in *PCCA*, and four novel mutations (c.1076 C > T, c.224 A > C, c.1339 C > T and c.1033G > C) were identified in *PCCB*. For detailed information, please refer to Table 4; Fig. 1.

**Table 4:** Genetic results of the patients and bioinformatic analysis of novel missense variants

Case	Clinical phenotype	Genetic characteristics						ExAC ALL	GnomAD ALL	SIFT	Polyphen2	Mutation Taster	REVEL	ACMG
		Gene	Allele origin	Variant location	Nucleotide	Novel variant	Variant type							
2	Late-onset	*PCCB*	P	E10	c.1076 C > T(p.P359L)	Y	M	-	-	D(0)	-	D(1)	D(0.89)	VUS
			M	E10	c.1087T > C(p.S363P)	N	M							
3	Late-onset	*PCCA*	P	E12	c.985T > A(p.Y329N)	Y	M	-	-	D(0)	-	D(1)	D(0.94)	LP
			M	E13	c.1195 C > T(p.R399W)	N	M							
4	Early-onset	*PCCB*	P	E2	c.224 A> C(p.D75A)	Y	M	-	-	D(0)	-	D(1)	D(0.99)	LP
			M	E13	c.1339 C> T(p.L447F)	Y	M	-	-	U(0.001)	-	D(1)	D(0.79)	P
6	Early-onset?	*PCCB*	P/M	E10	c.1033G > C(p.A345P)	Y	M	-	-	U(0.006)	-	D(1)	D(0.95)	LP

N: Nonsense; M: Missense. D: Deleterious; U: Uncertain; P: Pathogenic; LP: Likely pathogenic

### Pathogenicity prediction analysis

Variations c.985T > A in the *PCCA* gene and c.1076 C > T, c.224 A > C, c.1339 C > T and c.1033G > C in the *PCCB* gene are novel mutations not reported in the 1000 Genomes Project, gnomAD, or ExAC databases (Table 4). Missense mutations were analyzed using PolyPhen-2, MutationTaster, SIFT, and REVEL. All mutations were consistent with the patient’s clinical phenotype, which supports pathogenic evidence (Table 4).

**Fig. 2:**
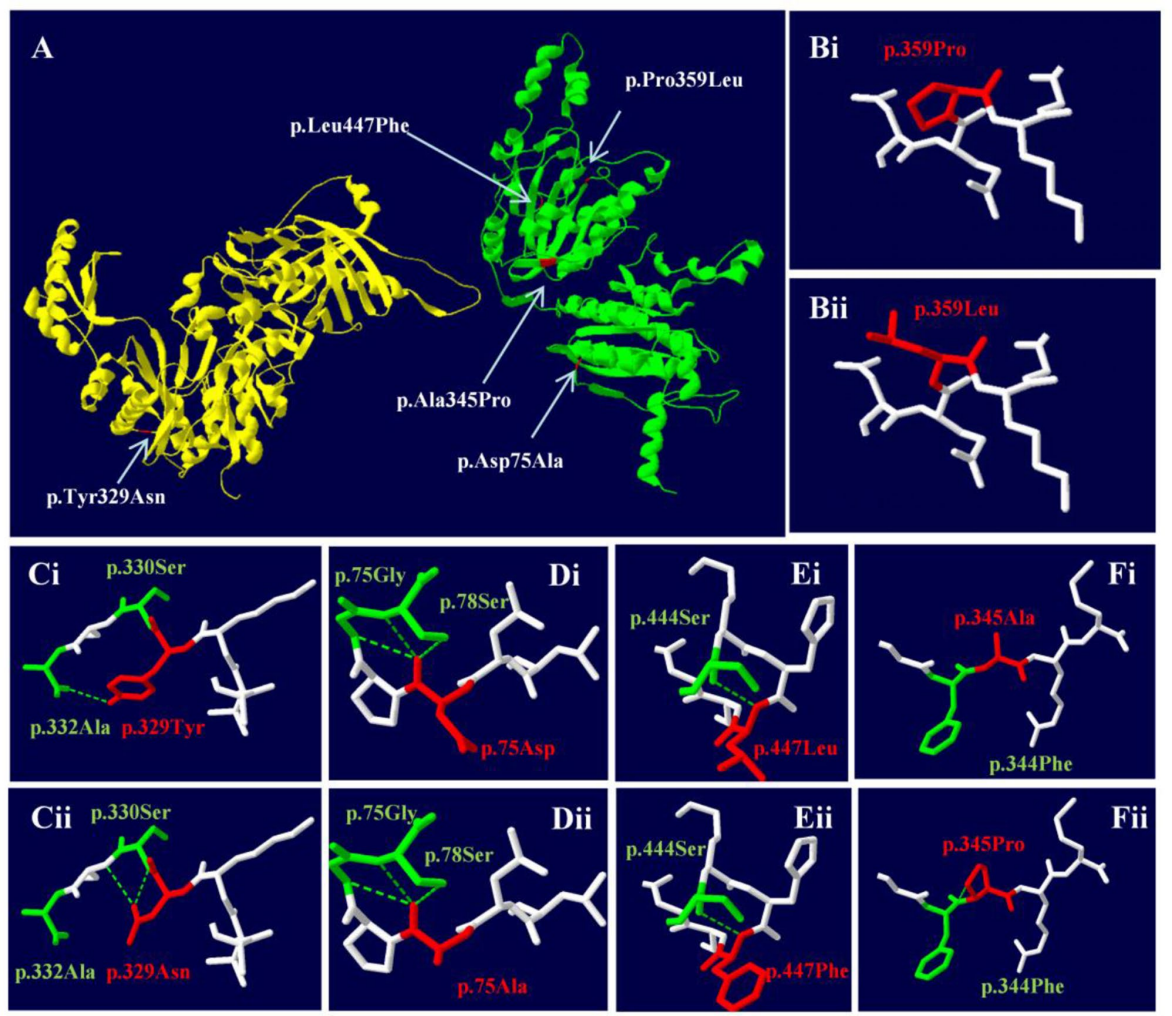
Three-dimension structure of *PCCA* and *PCCB* gene (wild type and mutant). (**A**) The three-dimension structure of the PA protein. The chain αwas highlighted in yellow, and the chain βin green. (**B**) The mutation changes the side chain structure. (**C**) The mutation makes it lose one hydrogen bond with alanine 332 and form a hydrogen bond with serine 331, which is expected to affect the stability of the protein structure. (**D**) The mutation changes the side chain structure. (**E**) The mutation changes the side chain structure. (**F**) The mutation leads to an additional hydrogen bond with phenylalanine 344

Swiss-PDB software was used to predict the effect of missense mutations on protein conformation in *PCCA* and *PCCB* (Fig. 2A). Mutations c.1076 C > T (p.P359L) (Fig. 2B), c.224 A > C (p.Asp75Ala) (Fig. 2D), and c.1339 C > T (p.Leu447Phe) in *PCCB* (Fig. 2E) altered the side chain structure of the protein. Mutations c.985T > A in *PCCA* (Fig. 2C) and c.1033G > C (p.Ala345Pro) in *PCCB* (Fig. 2F) affected both the side chain structure and hydrogen bond linkage with adjacent amino acids.

## Discussion

PA is a rare inherited metabolic disorder with high disability and mortality rates. We conducted a clinical and genetic study on six patients, broadening the spectrum of clinical phenotype spectrum of PA and the mutation spectrum of the *PCCA* and *PCCB* genes. We also explored the genotype-phenotype relationship in patients with PA. Despite the small sample size, our cases ranged from asymptomatic to death in the neonatal period, making them representative. In addition, we reported the updated incidence of PA in Beijing and Ningxia, China.

In our study, the incidence of PA in Beijing is 1/114,820, and in Ningxia is 1/189,671, which aligns with previously reported rates [15]. The overall incidence of PA in northern China was from 1/283,459 to 1/3,156 [14]. In Beijing, one patient carried *PCCB* gene mutations and one patient carried *PCCA* gene mutations. All patients (100%) in Ningxia carried *PCCB* gene mutations, which is inconsistent with a previous study where 24 (41.4%) patients had *PCCA* variants and 34 (58.6%) had *PCCB* variants [10]. This discrepancy may be due to the limited number of cases. We plan to continue studying the prevalence as our sample size increases.

The *PCCA* gene (OMIM 232000) is located on 13q32.3, contains 24 exons, and encodes 728 amino acids. The *PCCB* gene (OMIM 232050) is located on 3q22.3, contains 15 exons, and encodes 539 amino acids. They encode the α and β subunits of the mitochondrial biotin-dependent enzyme propionyl-CoA carboxylase, respectively [16]. The α subunit mainly contains three functional domains: the C-terminal biotin binding region, the biotin transfer region, and the N-terminal biotin carboxylase region. The β subunit hexamerizes with a propionyl-CoA binding site and a carboxyltransferase domain responsible for transferring the carboxyl group to propionyl-CoA [14]. Mutations in *PCCA* or *PCCB* genes lead to decreased PCC activity, causing PA. PA can be categorized into early-onset (< 3 months) and late-onset (> 3 months) forms [10]. Earlier onset is associated with more severe symptoms and worse prognosis. There is thought to be a connection between genotype and phenotype in PA patients, but further studies are needed [17]. The connection between gene mutation types, distribution, and phenotype remains unclear, but certain mutations may be related to the late-onset form or early-onset form of PA.

In our study, only c.1087T > C (p.S363P) in *PCCB* and c.1195 C > T (p.R399W) in *PCCA* were previously reported. The c.1087T > C (p.S363P) mutation in *PCCB* is related to the late-onset type [10]. The c.1195 C > T (p.R399W) mutation in *PCCA* is associated with a severe phenotype [18]. Additionally, we reported one novel mutation in the *PCCA* gene and six novel mutations in the *PCCB* gene. All these mutations have very low frequencies in the population and were predicted to be pathogenic by software analysis. In China, missense mutations are the most frequent type in *PCCA* and *PCCB*. In our study, 5 out of 7 (71.4%) were missense mutations.

The only patient (Case 3) with *PCCA* gene mutations had a late-onset form. All his blood and urine metabolic markers were only mildly elevated, and he had normal physical and mental development. He carries c.985T > A and c.1195 C > T, both in the biotin carboxylase region of PCCA. The latter is related to a severe phenotype, but given his mild clinical presentation, c.985T > A may be associated with a late-onset phenotype. Although his electrocardiogram and cardiac ultrasound results were normal, his CK and CK-MB levels were mildly elevated from 2 months of age, indicating that his cardiac function requires further follow-up.

Three patients (Cases 2, 4, and 6) carried *PCCB* gene mutations. Case 2 carries compound heterozygous mutations c.1076 C > T and c.1087T > C (p.S363P) in *PCCB*. The latter is a common mutation in Chinese PA patients and is related to the late-onset type, consistent with our findings [10]. Given his normal development, the novel mutation c.1076 C > T in *PCCB* may also be associated with late-onset. After treatment was discontinued, Case 4 died passed away at 3 months of age, suggesting that c.224 A > C (p.Asp75Ala) and c.1339 C > T (p.Leu447Phe) may be associated with early-onset. Case 5 and Case 6 are brothers, hypothesized to have identical genotypes and similar phenotypes. Case 5 had an early-onset form and died at 40 days old. Case 6 developed symptoms at 16 days of age and died at 18 months of age, and was found to carry homozygous mutations c.1033G > C (p.Ala345Pro) in *PCCB*. Based on the clinical presentation of these two cases, this mutation may be associated with an early-onset phenotype.

Early detection and treatment through newborn screening can improve the short-term prognosis. In our study, after one week of treatment, Case 4’s metabolic indicators improved significantly. His urine propionylglycine, 3-hydroxypropionic acid, methylcitric acid, and tiglylglycine decreased to near-normal levels, though methylcitric acid slightly decreased. His growth and development were normal until the treatment was discontinued. Case 5 and Case 6 are brothers and may carry the same mutations in the *PCCA* gene. Case 5 presented with drowsiness and feeding difficulties from 24 days old and died at 40 days old. However, his younger brother (Case 6) was called by the NBS center and accepted treatment at 11 days old. He died at 18 months of age. These cases indicates that early diagnosis and treatment can significantly improve short-term outcomes. However, in this study, early diagnosis and treatment through newborn screening did not result in an ideal long-term prognosis for patients with early-onset propionic acidemia. Current treatment mainly involves protein restriction [1], which fails to completely block the pathogenesis of PA or prevent poor prognosis. Some children still develop recurrent metabolic acidosis, cardiomyopathy, seizures, and developmental delays/intellectual disability [19, 20]. Therefore, studies related to longer follow-up and more effective treatments are needed to improve the long-term prognosis of PA patients.

In addition to metabolic disorders and neurological damage, myocardial involvement is a significant complication of PA. It can develop into arrhythmia, cardiomyopathy, severe heart failure, long-QT syndrome, or even cardiac arrest [21], which is life-threatening. Though Case 3 presented with slightly abnormal blood and urine metabolites and normal development, his CK and CK-MB were continuously elevated starting at 2 months old, implying that cardiac damage in PA patients may not be due to metabolic abnormalities alone but may involve other aspects of pathogenesis. His heart function needs long-term monitoring. Case 2 refused treatment. While he has not shown any intellectual developmental issues thus far, vigilance is required for potential cardiac complications or even the risk of sudden cardiac death. Due to the limited time and sample size of the study, we did not observe more complications. We will continue to expand the sample size and study time for further follow-up studies.

## Conclusion

The incidence of PA in Beijing is 1/114,820 and in Ningxia is 1/189,671. We reported six patients with PA and identified one novel mutation (c.1195 C > T) in the *PCCA* gene and four novel mutations (c.1076 C > T, c.224 A > C, c.1339 C > T, and c.1033G > C) in the *PCCB* gene. Mutations c.985T > A in *PCCA* and c.1076 C > T in *PCCB* may be associated with late-onset, while c.224 A > C, c.1339 C > T, and c.1033G > C in *PCCB* may be associated with early-onset.

### Limitation

Firstly, our study is limited by a small sample size, which may impact the robustness of genotype-phenotype correlation analyses. We will expand the sample size in further studies. Secondly, our follow-up time was limited. The long-term prognosis of PA patients with early diagnosis and treatment from neonatal screening remains unclear. We will conduct a longer-term follow-up study. Lastly, we were unable to perform functional experiments in this study. We aim to include these analyses in future research to deepen our understanding of the identified mutations.

## Data Availability

The raw sequence data reported in this paper have been deposited in the Genome Sequence Archive (Genomics, Proteomics & Bioinformatics 2021) in National Genomics Data Center (Nucleic Acids Res 2022), China National Center for Bioinformation / Beijing Institute of Genomics, Chinese Academy of Sciences (GSA-Human: HRA008005) that are publicly accessible at https://ngdc.cncb.ac.cn/gsa-human [22, 23].
